# An Approach for Reliably Investigating Hippocampal Sharp Wave-Ripples *In Vitro*

**DOI:** 10.1371/journal.pone.0006925

**Published:** 2009-09-07

**Authors:** Nikolaus Maier, Genela Morris, Friedrich W. Johenning, Dietmar Schmitz

**Affiliations:** 1 Neuroscience Research Center, Charité-Universitätsmedizin Berlin, Berlin, Germany; 2 Department of Neurobiology and Ethology, Faculty of Science and Science Education, Haifa University, Haifa, Israel; 3 Bernstein Center for Computational Neuroscience, Humboldt University Berlin, Berlin, Germany; Instituto Cajal - CSIC, Spain

## Abstract

**Background:**

Among the various hippocampal network patterns, sharp wave-ripples (SPW-R) are currently the mechanistically least understood. Although accurate information on synaptic interactions between the participating neurons is essential for comprehensive understanding of the network function during complex activities like SPW-R, such knowledge is currently notably scarce.

**Methodology/Principal Findings:**

We demonstrate an *in vitro* approach to SPW-R that offers a simple experimental tool allowing detailed analysis of mechanisms governing the sharp wave-state of the hippocampus. We combine interface storage of slices with modifications of a conventional submerged recording system and established *in vitro* SPW-R comparable to their *in vivo* counterpart. We show that slice storage in the interface chamber close to physiological temperature is the required condition to preserve network integrity that is necessary for the generation of SPW-R. Moreover, we demonstrate the utility of our method for studying synaptic and network properties of SPW-R, using electrophysiological and imaging methods that can only be applied in the submerged system.

**Conclusions/Significance:**

The approach presented here demonstrates a reliable and experimentally simple strategy for studying hippocampal sharp wave-ripples. Given its utility and easy application we expect our model to foster the generation of new insight into the network physiology underlying SPW-R.

## Introduction

A central characteristic of the hippocampus is its propensity to generate robust population rhythmic activity at various frequencies [1]–[3]. Among these, hippocampal sharp waves (SPWs) and associated ∼200 Hz ripples can be demonstrated in the EEG of resting subjects and have been implicated in the consolidation of recently acquired memories [2], [4]–[6]. In recent years, the application of multi-electrode recording and *in vivo* labeling techniques enabled the identification of the cell types involved in the SPW-R generating network [3],[7]–[9]. Based on these approaches, the temporal relations of these cells' firing were used to characterize the network mechanisms underlying ripples. However, comprehensive understanding of network function necessarily requires precise information of synaptic interactions among the participating neurons. Indeed, using *in vitro* models, several previous studies have focused on pharmacological and synaptic properties of sharp wave-ripples [10]–[23]. Technically, however, all these studies have been performed under experimental conditions that preclude targeted visual access to cells of interest, which has several advantages in comparison to blind patch- or sharp microelectrode recordings [24]–[26]. Here, we describe an approach to reliably studying SPW-R in hippocampal slices in the submerged condition *in vitro*.

We demonstrate the utility of our approach in presenting targeted whole-cell recordings during SPW-R. Taking full advantage of our system, we additionally demonstrate for the first time how the spatiotemporal dynamics of cells being recruited into the CA1 network during SPW-R can be studied using action potential-mediated Ca^2+^ transients at a single-cell resolution.

## Results

### Methodological requirements for SPW-R expression in submerged condition

**Figure 1:**
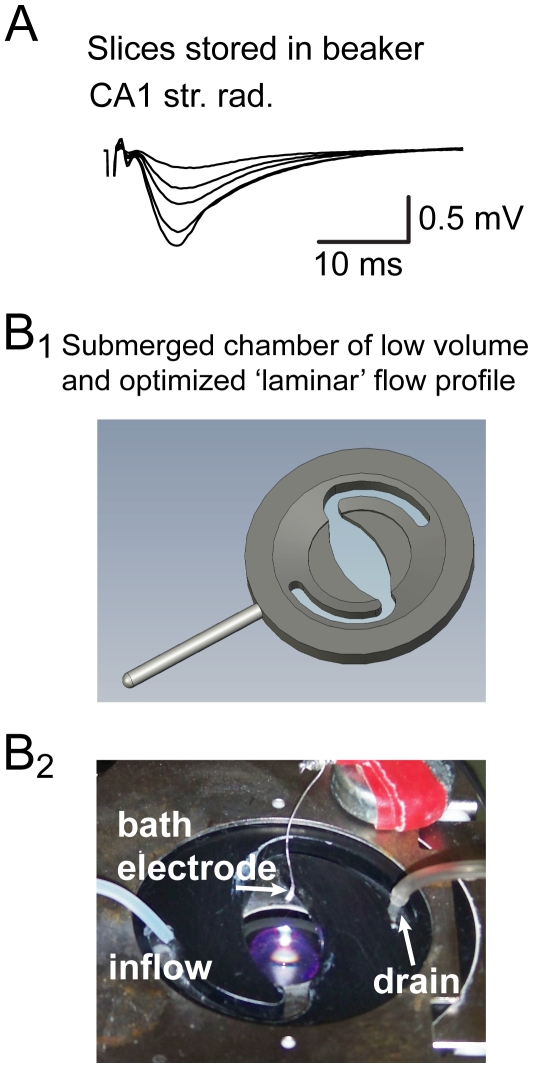
Experimental requirements for the reliable expression of sharp wave-ripples in submerged condition *in vitro*. A, slices stored in beakers and recorded from in conventional submerged chambers are viable but only anecdotally express sharp wave-ripples. Stimulus-induced field-EPSPs from these slices, however, revealed normal synaptic responses. 100 µs stimulation pulses of different intensities were applied at CA1 Schaffer collateral inputs. Stimulation artifacts are truncated. B1, submerged type recording chamber used throughout this study. The ovaloid shape of the chamber was chosen to enable flow conditions close to an ‘ideal’ laminar flow profile. B2, *‘in situ’* image of the used recording chamber. Str. pyr. and str. rad., stratum pyramidale and stratum radiatum, respectively (*n* = 48 slices for this study).

Our aim was to transfer our previously introduced model of SPW-R [10], [11] to the submerged-type electrophysiology setup to study the synaptic and network basis of SPW-R in a targeted way at the single-cell level. To that end, we recorded from slices in the standard submerged type electrophysiological recording chamber, after they had been conventionally stored in beakers filled with oxygenated ACSF [24]. Only episodically (1 out of 14 probed slices) we observed events in CA1 that resembled SPW-R. To control for possible general effects on the quality of slices under this condition we tested for viability of these slices by evoking stimulus-induced field-EPSPs, which could be successfully elicited under these conditions (Fig. 1A). Also, we found multi-unit bursting in area CA3 in all slices tested (*n* = 7; not shown). Together, though these slices exhibited normal electrophysiological properties, this approach did not provide a solid experimental course to study synaptic properties of SPW-R systematically *in vitro*.

For the second approach, we designed a submerged recording chamber facilitating ‘ideal’ flow-profile conditions, with small volume to enhance oxygen supply of the slices (∼1.6 ml; Fig. 1B1 and 1B2). For the same reason, we increased the perfusion rate of ACSF in our recording system (5–6 ml/min). Additionally, we switched to slices stored in an interface chamber, where slices are placed on the interface between gas (carbogen) and liquid (ACSF; 31–33°C, see Methods). This storage has been shown to promote the expression of *in vitro* SPW-R [10]–[12], [19]–[23], [27]–[29]. After having changed these experimental conditions, we observed SPW-R in the submerged setup in >90% of slices exhibiting these events in interface conditions (*n* = 48 slices for this study).

We wondered whether the temperature during slice storage is critical for the expression of sharp waves in submerged compared to interface maintenance. We therefore stored slices after otherwise identical preparation either in a beaker at 33°C, or in interface condition at room temperature. We found no SPWs in the slices stored in the interface condition at room temperature (*n* = 10). In 8 of the 10 probed slices stored in submerged condition at 33°C we observed signals that potentially corresponded to SPWs. However, these events had very small amplitudes (mean±SEM: 17.3±1.8 µV), were rare (0.27±0.06 Hz) and were not associated with any discernible oscillation. Based on these observations we conclude that the reliable expression of SPW-R *in vitro* relies on interface storage at near-physiological temperature.

### Storage type determines network excitability in CA3

**Figure 2:**
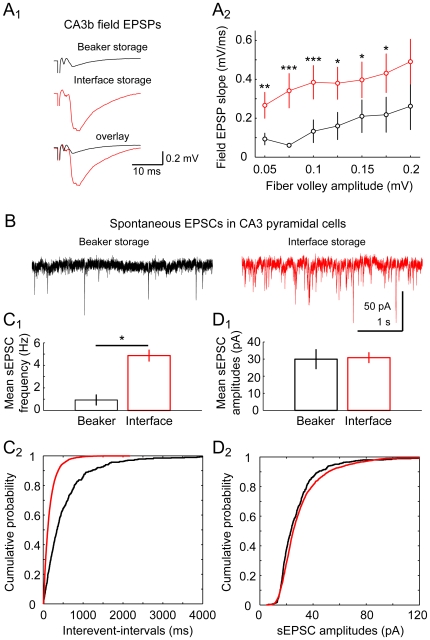
Slice storage determines excitability on the network level. Slices derived from identical preparations were stored in either interface chamber or beaker. A1, field responses in CA3b stratum radiatum were recorded upon stimulation of associational/commissural input. The afferent fiber volley was used as an independent measure of stimulation strength cross slices (see overlay for demonstration of fiber volley comparability). A2, comparison reveals larger field EPSPs in slices derived from interface storage (beaker, 6–11 slices and interface, 9–14 slices, respectively; levels of significance: * *P*≤0.05, ** *P*≤0.01, *** *P*≤0.001). B, in slices stored in beaker or interface, spontaneous EPSCs were recorded in CA3 principal neurons (1 µM gabazine present). Sample traces from both conditions are presented in black and red for beaker and interface storage, respectively. C1, analysis of mean sEPSCs incidence reveals a marked difference depending on storage condition (5 cells from each condition). Accordingly, comparison of cumulated inter-event intervals showed consistently higher values in cells recorded from beaker-stored slices (C2, two-sample Kolmogorov-Smirnov test; *P*<0.00001). D1, no significant change was found in averaged amplitudes of sEPSC (*P* = 0.7); comparison of amplitude distributions, however, revealed a small increase of sEPSC amplitudes in cells from interface-stored slices (D2, two-sample Kolmogorov-Smirnov test; *P* = 0.0001).

We next asked if the storage of slices influences excitability, thereby allowing or precluding the generation of sharp waves in hippocampal slices *in vitro*. We tested this hypothesis by probing slices from otherwise identical preparations that had been stored in either interface chamber or beaker. We concentrated on recordings from the CA3 area as this region has been demonstrated to be the initiation zone of sharp waves [1]. In a first set of experiments, we recorded CA3 network field responses following short current pulses delivered to the associational/commissural input (Fig. 2A1). Stimulation strength was controlled by monitoring the afferent fiber volley. We observed that field EPSPs had consistently lower voltages in slices stored in beaker (Fig. 2A2).

Bursting activity of CA3 principal neurons has been proposed to underlie the initiation of sharp waves [30]. Given our finding of reduced field EPSP amplitudes in beaker-stored slices we checked whether spontaneous excitatory network activity on the level of cellular recordings might also be different in slices stored in both conditions. To that end, we established voltage-clamp recordings in CA3 principal neurons in the presence of a GABA_A_ receptor antagonist (gabazine, 1 µM; −74 mV holding potential; Fig. 2B). Indeed, we observed a reduced incidence of spontaneous EPSCs (sEPSCs) in beaker-stored slices (beaker: 0.93±0.43 Hz; interface: 4.87±0.49 Hz; *P* = 0.008; 5 cells in both conditions; Fig. 2C1). Accordingly, we observed an increase in inter-event-intervals in sEPSCs from beaker-stored slices (*P*<0.00001; two-sample Kolmogorov-Smirnov test; Fig. 2C2). Additionally, though not changed on average (Fig. 2D1), the cumulative distribution of sEPSC amplitudes displayed a slight increase in this parameter in cells from interface- compared to beaker-stored slices (*P* = 0.0001; two-sample Kolmogorov-Smirnov test; Fig. 2D2). Together, these experiments suggest that the CA3 network is differently active depending on the storage system used for slice maintenance. Additionally, our results clearly show that interface storage is superior in the preservation of functional CA3 network in contrast to beaker-storage of slices. Therefore, interface-storage at near-physiological temperature is suggested to be the critical factor for expression of sharp wave-ripples in the *in vitro* slice preparation.

### Perfusion rate and recording temperature modulate SPW incidence

It has been proposed by other groups that elevated oxygen supply in the submerged recording system is the critical factor for expression of sharp wave-ripples [13], [14], [16]–[18], [28], [29]. To enhance oxygen availability these authors applied high perfusion rates and introduced elaborate perfusion systems that allows for oxygenation of both surfaces of the slice.

As we regularly use slices mounted on coverslips (see Methods), which precludes the oxygenation of the bottom of the slice, we hypothesized that double perfusion was not the critical parameter but might favor the expression of sharp waves *in vitro*.

**Figure 3:**
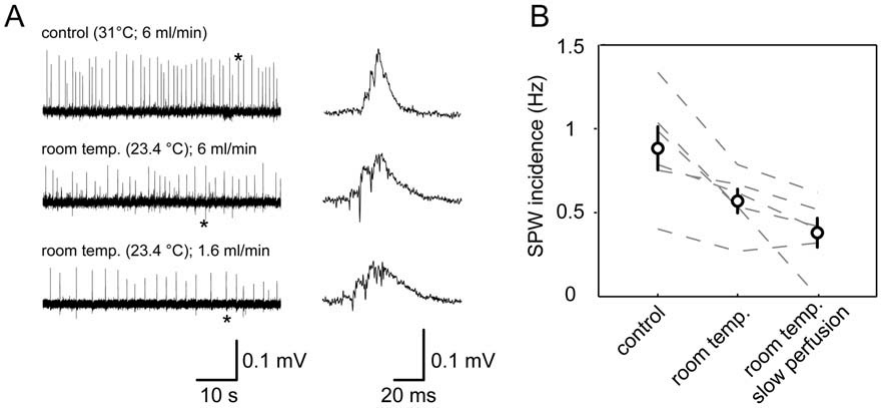
Perfusion rate and recording temperature modulate sharp wave incidence in submerged recordings *in vitro*. A, example experiment demonstrating the effect of successive reduction of recording temperature and perfusion rate on sharp wave occurrence. Magnified signals correspond to the events marked by asterisk. B, reduction of recording temperature and perfusion speed impairs but does not preclude the generation of sharp waves. Quantification was done 10–20 min after start of the respective experimental interference.

We therefore speculated that recording temperature and perfusion rate might be critical. In five experiments, we switched to room temperature during recordings. After 10–20 min under this condition, we indeed found a significant reduction of SPW incidence, which was even more pronounced when reducing the perfusion rate to 1.6 ml/min (control: 0.88±0.13 Hz; room temperature: 0.57±0.07 Hz; room temperature and slow perfusion rate: 0.38±0.09 Hz; *P* = 0.009, one-way ANOVA; Fig. 3). Notably, sharp waves could still be observed under these conditions, albeit with markedly reduced incidence.

Together, these experiments suggest that increased oxygenation by high ACSF perfusion rates, and physiological recording temperature are conditions favoring the generation of sharp waves *in vitro*; nonetheless, it is unlikely that these factors are the decisive determinants for SPW expression *in vitro*.

### Properties of sharp wave-ripples in submerged recording condition

**Figure 4:**
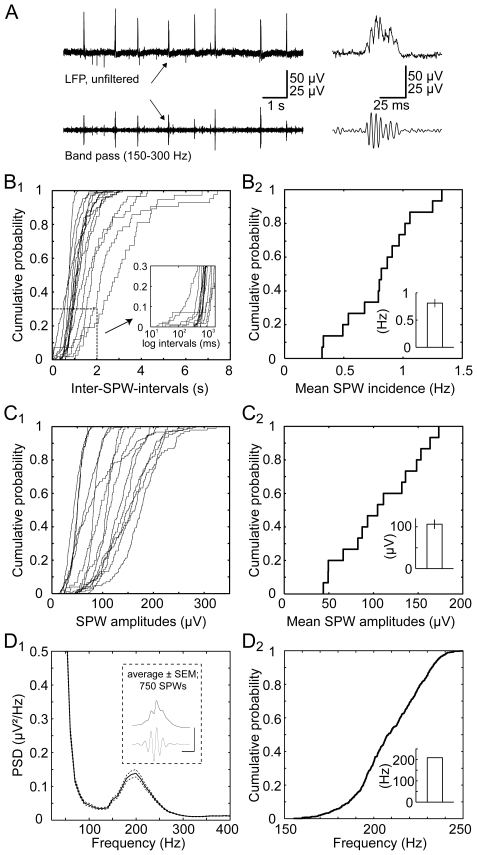
Basal properties of sharp wave-ripples in submerged recording conditions. A, example experiment of SPW-R sampled in CA1 stratum pyramidale (upper trace). Below: 150–300 Hz band pass-filtered derivatives of the above signal isolating the SPW-associated ripple oscillation. Right: temporally magnified trace segment as indicated by arrows. B1, as a representative sample, in 15 slices the incidence of sharp wave-ripples was determined. Cumulative probability plots show the individual distributions of inter-SPW-intervals as a measure of SPW incidence and illustrating within- and inter-slice variability. *Inset:* magnification of cumulative functions as indicated. B2, cumulative probability plot of mean values derived from the same 15 slices. *Inset* indicates mean incidence (0.81±0.08 Hz). C1, from the same pool of data SPW amplitudes were determined. The distributions of amplitudes from the individual experiments are presented, illustrating within- and inter-slice variability of this parameter. C2, mean SPW amplitudes cumulated from the same dataset. *Inset* represents the mean SPW amplitude (106.2±11.2 µV). D1, from the same pool of data, 50 sharp wave-ripple events were randomly chosen from each experiment. Power spectrum density (PSD) functions were computed on each of these 750 SPWs and averaged. Plot shows the averaged PSD function ± SEM. *Inset* shows peak-triggered SPW-average and its derived 150–300 Hz filtered ripple oscillation. Calibration: 100 and 25 µV; 10 ms. D2, on these PSD functions, frequency in the ripple frequency band was determined. Cumulative frequency distribution and mean value are shown (208.9±0.7 Hz, *inset*).

An example of sharp waves and associated ripples in our submerged approach is presented in Fig. 4A. We determined several basic properties of SPW-R in 15 slices. In each experiment, two-minute sample traces were analyzed. Figure 4B1 presents cumulative plots for the individual quantification of SPW incidence. Inter-SPW-intervals ranged from 17 ms to 7.4 s. On average, SPW incidence was 0.81±0.08 Hz (minimum: 0.32 Hz; maximum: 1.33 Hz; Fig. 4B2).

Similarly, we analyzed SPW amplitudes; cumulative results for individual slices are depicted in Fig. 4C1. SPW amplitudes ranged from 13.3 to 323.5 µV with an average of 106.2±11.2 µV (Fig. 4C2; quantification of mean amplitudes from 15 slices).

Ripples at ∼200 Hz have been demonstrated to be a hallmark of *in vivo* sharp waves [31]–[34]. To check for a similar feature of SPWs using our submerged *in vitro* approach, we analyzed the spectral properties of sharp wave-ripples in our experimental system and indeed identified a clear peak at ∼200 Hz in all the power spectra, consistent with sharp wave-associating ripples *in vivo*. Figure 4D1 displays the average of all power spectrum density (PSD) plots of the 750 single sharp wave-ripple events analyzed. Quantification of the center of mass of the individual PSD functions revealed a mean ripple oscillation frequency of 208.9±0.7 Hz (see Fig. 4D2 for the respective cumulative frequency distribution).

### Spatial characteristics of sharp wave-ripples in submerged condition

**Figure 5:**
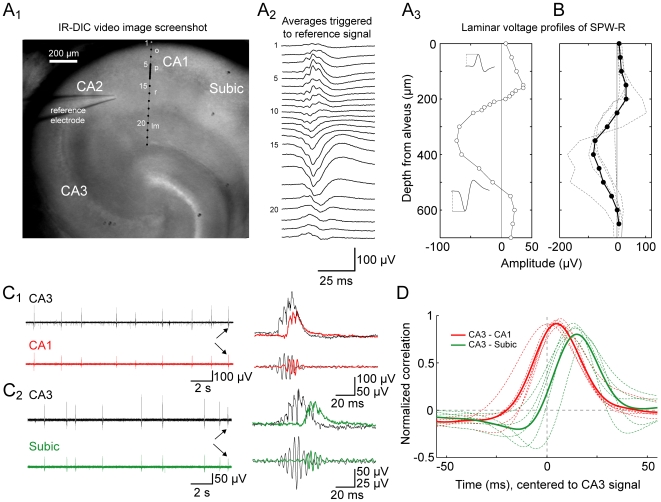
Spatial characteristics of sharp wave-ripples in submerged recordings. A, serial multisite LFP recordings were performed to reveal the spatial profile of sharp wave-ripples in CA1. A1, infrared-differential contrast (IR-DIC) video image displaying the reference electrode positioned in CA1 stratum radiatum close to CA2 while SPW-R were recorded with a second electrode in CA1 at locations closer to the subiculum (from right, positions as indicated, 10 to 100 µm steps). O, stratum oriens; p, stratum pyramidale; r, stratum radiatum; lm, stratum lacunosum-moleculare; Subic, subiculum. A2, voltage profile of SPW-R of the experiment shown in A1. LFP averages, each representing 20 events, were triggered by the signal sampled with the reference electrode. A3, amplitude depth profile of the example experiment shown in A1-A2. At each recording position with increasing distance to the alveus, the first significant maximum from baseline was determined as the peak of the SPW (see insets for examples regarded as positive and negative events). B, averaged amplitude depth profile (*n* = 5, solid line) and single depth profile functions (dashed). C, paired LFP recordings in CA3 and CA1 (C1) or subiculum (C2) of the same slice (red and green, respectively). Right: overlay of the indicated events and corresponding ripple signals (150–300 Hz) below. Note the increments of delays of CA1 and subiculum events *versus* SPWs recorded in CA3. D, normalized crosscorrelation functions to quantify latencies of SPW-R sampled from CA1 and subiculum with respect to the simultaneously recorded reference signal from CA3 (single experiments, dashed; average, bold; red, CA1 (*n* = 6); green, subiculum (*n* = 5)). Note increase in latency of subicular *versus* CA1 sharp waves, representing the spread of SPW-R towards the output structures of the hippocampus.

We next aimed at studying further properties of sharp waves in our submerged approach. We evaluated the spatial distribution of *in vitro* SPW-R, i.e. their amplitudes over somato-dendritic recording positions in area CA1. Guided by the infrared differential interference contrast (IR-DIC) video image we recorded SPWs from up to 32 recording sites in 10–100 µm steps starting from the alveus (Fig. 5A1). Similar to the initial comprehensive description of SPWs in dorsal hippocampus *in vivo* by Buzsáki (1986), in our approach in slices from ventral hippocampi we observed positive voltages in the CA1 pyramidal cell layer and prominent negative amplitudes in stratum radiatum (Fig. 5A2,A3 and B).

Sharp waves *in vivo* emerge in CA3 and propagate towards CA1 and the subiculum [1], [30]. We were able to demonstrate a similar propagation of SPWs in our *in vitro* system. In paired LFP recordings we sampled SPW-R from CA3 and CA1 or subiculum and evaluated their temporal relations. Indeed we observed a highly correlated occurrence of SPWs in CA3 and downstream areas (Fig. 5C1 and 5C2). Moreover, correlation functions of CA3–subiculum recordings displayed increased latencies compared to those of CA3–CA1 samples (mean latencies, 15 ms *versus* 5 ms; Fig. 5D). It has to be noted that subicular sharp waves in our submerged approach are less reliably expressed than their CA3 and CA1 counterparts.

Together, these findings on spatial properties of SPW-R in our *in vitro* approach are well comparable with spatial characteristics of SPWs *in vivo* [1], [30], [33], [34].

### Targeted recordings from neurons during sharp wave-ripples

Using juxtacellular recordings in anaesthetized rats, Klausberger and co-workers previously demonstrated the differential activation of hippocampal cells during sharp wave-ripples [3],[7]–[9]. This technique, however, restricts the analysis to suprathreshold, *i.e.* to active spiking behavior of participating neurons. We demonstrate that our *in vitro* approach to SPW-R complements the *in vivo* approach in that it enables the targeted exploration of subthreshold postsynaptic activity in cells that make up the network.

**Figure 6:**
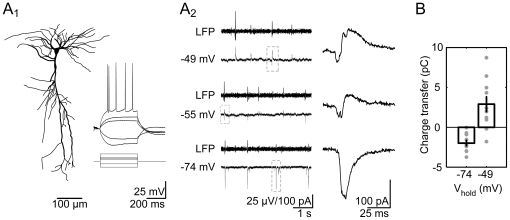
Targeted patch-clamp recordings from CA1 principal cells during SPW-R. A1, Pyramidal neurons of area CA1 were characterized electrophysiologically and labeled with biocytin for reconstruction. Injected current steps for characterization correspond to −200, −80, 80, 120, 160 and 280 pA. A2, dual LFP- and close-by (<100 µm) whole cell patch clamp recording during SPW-R. Amplitude and polarity of compound SPW-R-coupled postsynaptic currents depend on the holding potential. Magnified events (right panel) are indicated by boxes (left panel). B, analysis of total charge transfer close to RMP (−74 mV) and depolarized holding potential (−49 mV; 10 cells analyzed).

To demonstrate this advance, we recorded from CA1 principal neurons (e.g., Fig. 6A1). We established whole-cell voltage-clamp recordings in cells that were at close proximity to the LFP recording electrode (<100 µm), as determined by visual inspection on the IR-DIC video image. Several basic intracellular properties were analyzed, including the cell's resting membrane potential (RMP), input resistance (R_i_), action potential (AP) amplitude, and width at half peak. We verified values typical of CA1 pyramidal cells (RMP: −76.9±1.8 mV; R_i_: 193.9±35.2 MΩ; AP amplitude: 105.5±3.3 mV, and AP width: 1.3±0.01 ms, respectively; 10 cells). All SPWs observed in the extracellular recording were associated with compound postsynaptic currents in pyramidal cells. To elucidate the synaptic currents contributing to SPW-R, we systematically varied the holding potential (Fig. 6A2). We calculated the theoretical reversal potential for chloride (∼−67 mV, see Methods). At ∼−74 mV we observed prominent inward postsynaptic currents during LFP sharp waves. Conversely, at more depolarized levels (∼−49 mV), we found inward currents followed by more prominent outward currents, indicating an initial depolarization followed by large inhibition. On average, total charge transfer during these compound SPW-associated currents was −1.98±0.37 pC at ∼−74 mV and 2.90±1.01 pC at ∼−49 mV (10 cells; Fig. 6B).

Together, as demonstrated here, our *in vitro* approach to sharp wave-ripples enables IR-DIC video microscopy-based targeted recordings from identified neurons and hence offers a comparably easy way to investigate subthreshold synaptic activity that is present during ripple oscillations.

### Fluorescence Ca^2+^ imaging facilitates characterization of SPW-R on the single cell- and network level

**Figure 7:**
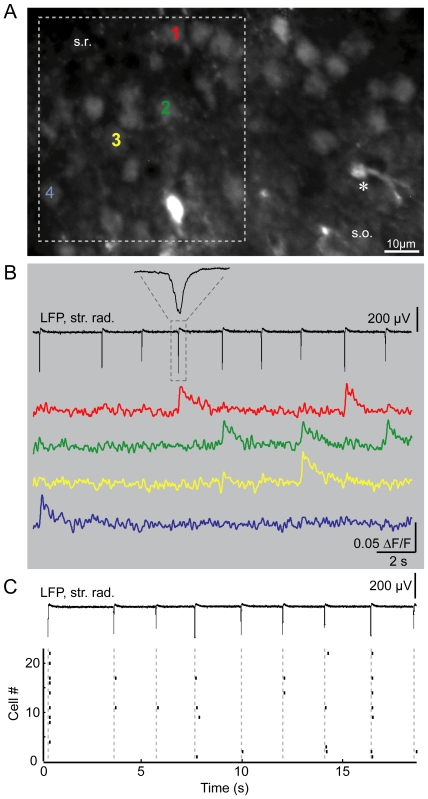
Population imaging of somatic Ca^2+^ transients during sharp wave-ripples. A, high-resolution overview image of an OregonGreen-BAPTA1 bulk-loaded group of cells in the CA1 pyramidal layer of the hippocampus. The white box refers to the subregion imaged for the time-lapse recordings. Compared to the overview picture, Ca^2+^ measurements were performed on a smaller number of cells limited by the size of the camera chip used for Ca^2+^ imaging. Single cells can be well distinguished. The asterisk indicates a putative astrocyte. B, LFP recording from *stratum radiatum* (str. rad.) and corresponding time courses of the somatic Ca^2+^ signals indicative of suprathreshold activation and subsequent AP firing from cells labelled 1–4 in A. C, raster plot of significant responses imaged in A. Grey dashed lines correspond to peak negativities of sharp waves. Note that significant somatic Ca^2+^ signals indicative of cellular spiking can be exclusively found temporally coupled to sharp waves.

Another advantage of our approach is that it enables simultaneous investigation of SPW-R and Ca^2+^ signals at the single cell- and network level. In principle, submerged conditions permit the use of water immersion objectives with high numerical apertures, which are a prerequisite for combining high-resolution imaging in acute brain slices with cellular electrophysiology. Using bulk loading of fluorescent Ca^2+^ indicators, action potential-mediated Ca^2+^ transients can be recorded simultaneously from large neuronal populations with strict single-cell resolution. Depending on the magnification and size of the CCD camera sensor, this method can be extended to the simultaneous recording of thousands of neurons [35]. In Fig. 7, we present an example of how high-speed time lapse imaging of somatic Ca^2+^ transients can be combined with LFP recordings from SPWs. In this example, a ventral horizontal slice was chosen resulting in the scattered appearance of the CA1 *stratum pyramidale*. From these data, we were able to reconstruct the spatiotemporal dynamics of SPW-associated spiking in single cells (Figs. 7B–C). The average amplitude of the detected events was 0.03±0.002 ΔF/F. 12 of the 22 recorded cells responded during sharp wave activity. During the 9 sharp waves imaged here, we detected 31 events, so an average of 3.4 cells responded per sharp wave.

## Discussion

In this study we have described an experimentally simple *in vitro* approach that allows studying hippocampal sharp wave-ripples in the standard submerged-type electrophysiological setup. By comparing excitatory network activities on the field- and single cell level in slices from two storage systems we showed the predominance of interface- over beaker storage for the preservation of network function that is required for SPW generation. Analysis of SPW-R in our submerged approach revealed that they are phenomenologically similar to sharp wave-ripples *in vivo* with respect to spectral characteristics, spatial profile and propagation through the hippocampal network. Additionally, we have shown the experimental utility of our approach in that it allows for targeted recordings of involved cells by visual guidance of IR-DIC microscopy; also, we have demonstrated the model's benefit in allowing the investigation of spatiotemporal cellular activation patterns using Ca^2+^ imaging during SPW-R.

A methodologically important result of our study is that slice storage in the interface chamber enhances the excitability of the CA3 network compared to the conventional submerged stor-age in ACSF-filled beaker. Schuchmann et al. have directly compared extracellular space volume of brain tissue in these two maintenance systems [36]. Indeed, for hippocampal slices, they demonstrated that the interface condition leads to a significant reduction of extracellular space volume compared to the submerged condition. The mechanistic link between reduced extracellular space volume and increased synaptic excitability remains to be elucidated. It might be speculated that reduced extracellular space volume contributes to the preservation of the neuronal network architecture after the slicing procedure thus promoting the reliable expression of sharp wave-ripples in slices stored in the interface chamber.

Several other groups have studied sharp waves in submerged recording systems previously. For instance, Wu and colleagues have demonstrated that the intact hippocampal isolate maintained in submerged conditions at high perfusion rate (∼15 ml/min) expresses spontaneous rhythmic field potentials reminiscent of sharp waves *in vivo* [14]. In other studies, these colleagues have refined their approach in introducing the 500–800 µm ‘thick slice’ preparation for studying sharp waves *in vitro* [13], [15]–[18], [29]. More recently, Hájos and co-workers described spontaneous sharp waves occurring in slices maintained in slightly modified ACSF [28]. In line with our approach, they used the interface chamber to store slices prior to recordings in the submerged setup. In their hands, though, stability of oscillation generation and SPW-R propagation from CA3 to CA1 was only achieved when slices where dually superfused from both surfaces.

In contrast to these studies, our approach, using slices of 400 µm in standard, non-modified ACSF, does not require double superfusion while reliably expressing sharp wave-ripples. Importantly, the use of polylysine-coated coverslips to attach the slice to the floor of the recording chamber enables us to visualize target cell types of interest via the infrared DIC video microscope. This advance will also enable the use of genetically engineered animals that express fluorescent proteins in specific cells [37] for the study of synaptic properties during sharp wave-ripples. Further, our approach offers the opportunity to apply Ca^2+^ imaging techniques during SPW-R. Indeed, to the best of our knowledge, our study comprises the first direct demonstration of suprathreshold neuronal activation patterns of the hippocampal network using Ca^2+^ signals during SPW-R as readout. The scattered spatial and temporal distribution of AP-mediated Ca^2+^ signals in the observed cell population contrasts the homogenous activation pattern of inward/outward current sequences observed when using whole-cell patches in the voltage clamp mode. However, the erratic activation pattern of cells in the absence of membrane voltage control can be explained by the strong inhibition following initial depolarizing currents, which limits the probability of all-or none spiking responses.

In summary, the approach we presented here offers an experimentally simple strategy to study hippocampal sharp wave-ripples in a reliable fashion, on the network- and single-cell level. Given its easy application and experimental utilities our approach will contribute to future experiments further deciphering the cellular, synaptic and network bases of hippocampal SPW-R.

## Materials and Methods

### Ethics Statement

Animal husbandry and experimental intervention was performed according to the German animal welfare act and the European Council Directive 86/609/EEC regarding the protection of animals used for experimental and other scientific purposes. All animal maintenance and experiments were performed in accordance with the guidelines of local authorities, Berlin (T0100/03).

### Slice preparation

C57Bl/6 mice of both sexes (4–8 weeks) were anesthetized with an isoflurane-vaporiser and decapitated. Brains were transferred to cooled (1–4°C) standard artificial cerebrospinal fluid (ACSF), containing (mM): NaCl 119, KCl 2.5, MgCl_2_ 1.3, CaCl_2_ 2.5, glucose 10, NaH_2_PO_4_ 1.0, NaHCO_3_ 26, gassed with carbogen (95% O_2_/5% CO_2_; pH 7.4 at 37°C; 290–310 mosmol/l). Horizontal slices (400 µm) of the ventral hippocampus were cut on a microslicer (VT1200 S; Leica, Germany), and stored in submerged [24] or interface conditions. A modified Haas-type interface chamber [38] was used, allowing us to maintain up to 10 slices in a 20×45×8 mm storage container. Slices were held at 31–33°C, continuously oxygenized with carbogen, and superfused with ACSF at 1 ml/min. Slices were allowed to recover for at least 2 hours after slicing. For recordings, we mounted slices on polylysine-coated coverslips (see below) and transferred them to the submerged-type recording chamber.

### Electrophysiology

Recordings were performed in standard ACSF at 32°C in a submerged modified recording chamber perfused at high rate (5–6 ml/min; see below). Glass microelectrodes (tip diameter ∼5–10 µm; resistance: 0.2–0.3 MΩ) were filled with ACSF before use. Extracellular signals were amplified 1000-fold, filtered (1–2000 Hz) and sampled at 5 kHz.

Whole-cell recordings were performed using a Multiclamp 700A amplifier (Axon Instruments, Union City, USA). Borosilicate glass electrodes (2–5 MΩ) were filled with (mM): K-gluconate 120, KCl 10, Hepes 10, Mg-ATP 3, EGTA 5, MgSO_4_ 2, GTP 1; pH was adjusted to 7.2 with KOH.

Principal cells were identified using infrared differential interference-contrast (IR-DIC) video microscopy. In the whole-cell configuration, de- and hyperpolarizing current steps (200–1000 ms) were applied to characterize the cell's intrinsic properties; only cells that showed typical spiking characteristics of principal neurons were considered. Series resistance R_s_ was monitored continuously throughout experiments; cells were rejected if R_s_ was >20 MΩ or varied >±30% during recordings. No R_s_ compensation was used. Cellular potentials indicated are liquid-junction potential-corrected (calculated ∼14 mV). The reversal potential of chloride was determined applying the Nernst equation based on the extra- and intracellular concentrations in our experiments (129.1 mM and 10 mM, respectively). Cells were routinely loaded with 0.3–0.5% biocytin.

### Ca^2+^ imaging

Multi-cell bolus loading of slices was performed as described elsewhere [39]. Oregon Green BAPTA1-AM was used at 500 µM. Bolus application resulted in a 200×200 µm loading spot. For time-lapse confocal recordings, a Yokogawa CSU-22 spinning disc confocal system (BFI Optilas, Puchheim, Germany) was coupled to an Olympus BX-51WI upright microscope and a RedShirt NeuroCCD-SMQ camera (Life Imaging Services, Reinach, Switzerland). Imaging with the spinning disc confocal microscope was limited to superficial layers of the slice. Excitation was provided at 488 nM by a Coherent Sapphire 488-50 Laser (Coherent, Utrecht, Netherlands). Using the Olympus XLumPlan Fluorit 20×0.95NA water immersion objective, the lateral pixel size was 1.2 µm. Full frames were recorded at 125 Hz. For high-resolution images, we used a Lumenera Infinity 2-1 camera (BFI Optilas, Puchheim, Germany) with a larger field of view. Therefore, only a subset of the cells displayed in the overview imaging was used for Ca^2+^ imaging.

### Data analysis

All analysis was done using Matlab, The Mathworks, (Aachen, Germany). *Identification of SPW-R events*. Recordings in the pyramidal cell layer reveal SPW-R that appear as positive deflections in the LFP. These events occur with a frequency of ∼0.7–1 Hz. Therefore, the vast majority of LFP samples (at a 5000 Hz sampling rate) belong to the background noise. In particular, the mode of the LFP distribution is a good central statistic of the noise. We estimated baseline noise by fitting a normal distribution to all samples smaller than the mode, and their reflection with respect to the mode. A threshold was set at 3.5 STD, and all samples larger than this threshold were considered as possible events. Since events typically last 50–150 ms, we introduced an additional length criterion. From the above candidates, only events in which a continuous deflection of over 1 STD from baseline lasted for >20 ms were considered SPW-R events. Peak time and amplitude of each such event was recorded for further analysis of intracellular and extracelluar data. A subset of the data underwent a manual identification process. In this method, the same procedure as described above was applied in the first stage, but with a lower threshold of 1.5 STD. All events that passed this threshold and the additional length criterion were displayed on a PC screen. The user could then accept the event as a SPW-R or reject it. A subset of experiments underwent both automatic and manual event identification, to verify our method. Comparison of both ways of detection revealed identical results.

*Spectral analysis* of sharp wave-ripples was computed with the Fast Fourier Transform algorithm applied on stretches of 100 ms of raw data centered on the SPW peak. Frequency resolution of the resulting power spectrum density (PSD) plots was 9.8 Hz. *Power* in the ripple band was determined by integrating individual PSD functions between 120 and 300 Hz. The mean *ripple frequency* was identified by the center of mass of the PSDs in that frequency range.

*AP height* was determined as the voltage difference between RMP and AP maximum. Correspondingly, we computed AP half-width as the temporal difference at the crossing times of 50% of AP height.

To quantify *charge transfer* in SPW-associated postsynaptic currents we analyzed traces of 100 ms windows surrounding the peak of the extracellularly identified SPW-R. Charge transfer was determined as the time integral of currents in these windows.

*Ca^2+^-mediated changes in fluorescence* over time were measured in the CA1 pyramidal cell layer. The resulting traces were Gaussian-filtered. For raster plots, responses were accepted when the peak amplitude was >2 * SD of baseline noise.

Data are presented as means ± SEM. Unless otherwise stated, statistical significance was assessed using Wilcoxon's rank sum test at the given significance level (*P*).

### Slice processing and anatomical reconstruction

After recording, slices were transferred to a fixative solution containing 4% paraformaldehyde and 0.2% saturated picric acid in 0.1 M phosphate buffer. Slices were re-sectioned into 70 µm thin sections. Biocytin-filled cells were subsequently visualized with 3,3′-diaminobenzidine tetrahydrochloride (0.015%) using a standard ABC kit (Vectorlabs, Burlingame, CA, USA) and reconstructed on a light microscope at 40× with a Neurolucida 3D reconstruction system (MicroBrightField, Williston, VT, USA).

### Polylysine coating of coverslips

Glass coverslips (10 mm diameter) were cleaned with 1 N HCl in an ultrasonic bath for 15 minutes, rinsed in de-ionized water, and cleaned in de-ionized water in the ultrasonic bath for another 3×15 minutes. Afterwards, coverslips were kept in de-ionized water for 24 hours followed by storage in 96% ethanol for at least three days. For coating, coverslips were removed from ethanol and dried. Stock solution of poly-d-lysine hydrobromide (1 mg/ml H_2_O, stored at 4°C) was diluted with de-ionized water (1∶10), drops of ∼100 µl were mounted on the coverslips that were dried overnight.

### Applied drugs

2,3-dioxo-6-nitro-1,2,3,4-tetrahydrobenzo(f)quinoxaline-7-sulfonamide (NBQX) and poly-d-lysine hydrobromide were purchased from Sigma Aldrich, Germany, 6-Imino-3-(4-methoxyphenyl)-1(6H)-pyridazinebutanoic acid hydrobromide (gabazine) was obtained from Biotrend, Germany, and Oregon Green BAPTA1-AM from Invitrogen, Germany.
